# Calmodulinopathy: A Novel, Life-Threatening Clinical Entity Affecting the Young

**DOI:** 10.3389/fcvm.2018.00175

**Published:** 2018-12-06

**Authors:** Maria-Christina Kotta, Luca Sala, Alice Ghidoni, Beatrice Badone, Carlotta Ronchi, Gianfranco Parati, Antonio Zaza, Lia Crotti

**Affiliations:** ^1^Istituto Auxologico Italiano, IRCCS, Center for Cardiac Arrhythmias of Genetic Origin and Laboratory of Cardiovascular Genetics, Milan, Italy; ^2^Department of Biotechnology and Bioscience, University of Milano-Bicocca, Milan, Italy; ^3^Department of Medicine and Surgery, University of Milano-Bicocca, Milan, Italy; ^4^Istituto Auxologico Italiano, IRCCS, Department of Cardiovascular, Neural and Metabolic Sciences, San Luca Hospital, Milan, Italy

**Keywords:** CALM, calmodulin, long QT syndrome, sudden cardiac death, catecholaminergic polymorphic ventricular tachycardia

## Abstract

Sudden cardiac death (SCD) in the young may often be the first manifestation of a genetic arrythmogenic disease that had remained undiagnosed. Despite the significant discoveries of the genetic bases of inherited arrhythmia syndromes, there remains a measurable fraction of cases where in-depth clinical and genetic investigations fail to identify the underlying SCD etiology. A few years ago, 2 cases of infants with recurrent cardiac arrest episodes, due to what appeared to be as a severe form of long QT syndrome (LQTS), came to our attention. These prompted a number of clinical and genetic research investigations that allowed us to identify a novel, closely associated to LQTS but nevertheless distinct, clinical entity that is now known as *calmodulinopathy*. Calmodulinopathy is a life-threatening arrhythmia syndrome, affecting mostly young individuals, caused by mutations in any of the 3 genes encoding calmodulin (CaM). Calmodulin is a ubiquitously expressed Ca^2+^ signaling protein that, in the heart, modulates several ion channels and participates in a plethora of cellular processes. We will hereby provide an overview of CaM's structure and function under normal and disease states, highlighting the genetic etiology of calmodulinopathy and the related disease mechanisms. We will also discuss the phenotypic spectrum of patients with calmodulinopathy and present state-of-the art approaches with patient-derived induced pluripotent stem cells that have been thus far adopted in order to accurately model calmodulinopathy *in vitro*, decipher disease mechanisms and identify novel therapies.

## Introduction

Sudden cardiac death (SCD) is an unexpected natural death due to cardiac causes that is responsible for up to 25% of all deaths in the Western world (1). While SCD as a term describes a final common clinical outcome, it does not by itself relay information on the pathophysiological mechanisms underlying its occurrence, which are quite distinct, especially when considering the age of the SCD victim. Indeed, in the young (< 35 years), SCD is mainly the adverse outcome of inherited cardiac diseases, such as cardiomyopathies and channelopathies, with ventricular fibrillation (VF) being mostly the culprit arrhythmia (2). Unfortunately, SCD may afflict even those in the perinatal, neonatal and early childhood period. We and others have shown that when post-mortem clinical investigations fail to identify the underlying causes (“mors sine materia” or “normal heart” SD) (3), channelopathies, and the long QT syndrome (LQTS) in particular, may contribute significantly to cases of sudden infant death syndrome (4), as well as intrauterine fetal death and stillbirth (5). In these cases, early-onset and highly malignant arrhythmias caused by penetrant genetic mutations in ion channel genes, or their accessory protein partners, often arising *de novo*, largely dictate SCD occurrence.

A few years ago, two infants having experienced recurrent VF episodes and suffering from what appeared to be as an unusually severe form of LQTS (QTc > 600 ms, intermittent 2:1 atrioventricular block and T wave alternans) came to our attention. Both were born to healthy parents and genetic testing of the major LQTS genes was negative. In order to identify what we presumed to be a novel underlying genetic cause of these severe arrhythmia manifestations, we performed whole-exome sequencing in both infants (as part of parent-child trios) and identified novel mutations in two of the three genes encoding the Ca^2+^-signaling protein calmodulin (*CALM1*-p.D130G and *CALM2*-p.D96V) that were shown to have arisen *de novo*, thus explaining the parents' normal phenotype (6). This finding was further validated by expanding our search for calmodulin (CaM) mutations in a pool of unrelated LQTS patients that were genetically negative for mutations in the main LQTS genes. In doing so, we identified the same *CALM1*-p.D130G mutation as well as the novel *CALM1*-p.F142L mutation in two other unrelated LQTS cases with severe disease and recurrent cardiac arrest episodes (6). Shortly after, other investigators identified the same p.D130G mutation, albeit in the *CALM3* gene, as a novel genetic substrate of severe LQTS (7), thus completing the picture of a “genetic trilogy” for a new clinical entity that has been termed *calmodulinopathy* (8).

In the past few years our knowledge on calmodulinopathy has expanded and it is now known to be a severe arrhythmogenic condition that can manifest mainly as LQTS (6), catecholaminergic polymorphic ventricular tachycardia (9) or idiopathic VF (IVF) (10) caused by genetic mutations in any of the 3 calmodulin genes (*CALM1, CALM2, CALM3*). Calmodulin is a ubiquitously expressed protein that participates in a plethora of cellular processes, while acting intracellularly both as a Ca^2+^ sensor and signal transducer. In the heart, CaM is a major player in the modulation of several ion channels such as the L-type calcium channel (LTCC), the sodium channel, different potassium channels, and the ryanodine receptor (RyR) (11).

In this review, we will provide an overview of currently available knowledge on this severe arrhythmogenic syndrome, termed calmodulinopathy. In particular, we will present in detail CaM's sequence, structure and function, the genetic spectrum of CaM mutations and the associated phenotypes, as well as available therapies. We will also overview the underlying disease mechanisms of calmodulinopathy (reviewed in detail in the accompanying article by Badone et al.) and present the thus far used *in vitro* methods for deciphering these disease mechanisms.

## Calmodulin Genes and Protein

The first complementary DNA (cDNA) clone of human CaM was isolated from a human liver cDNA library in 1984 (12). Within a few years, two more human cDNA clones were isolated and characterized (13, 14) with diverse nucleic acid sequence identity but all encoding an identical CaM protein. These results suggested the existence of a multigene CaM family operating under selective pressure to regulate and express a protein with a prominent cellular role.

**Figure 1:**
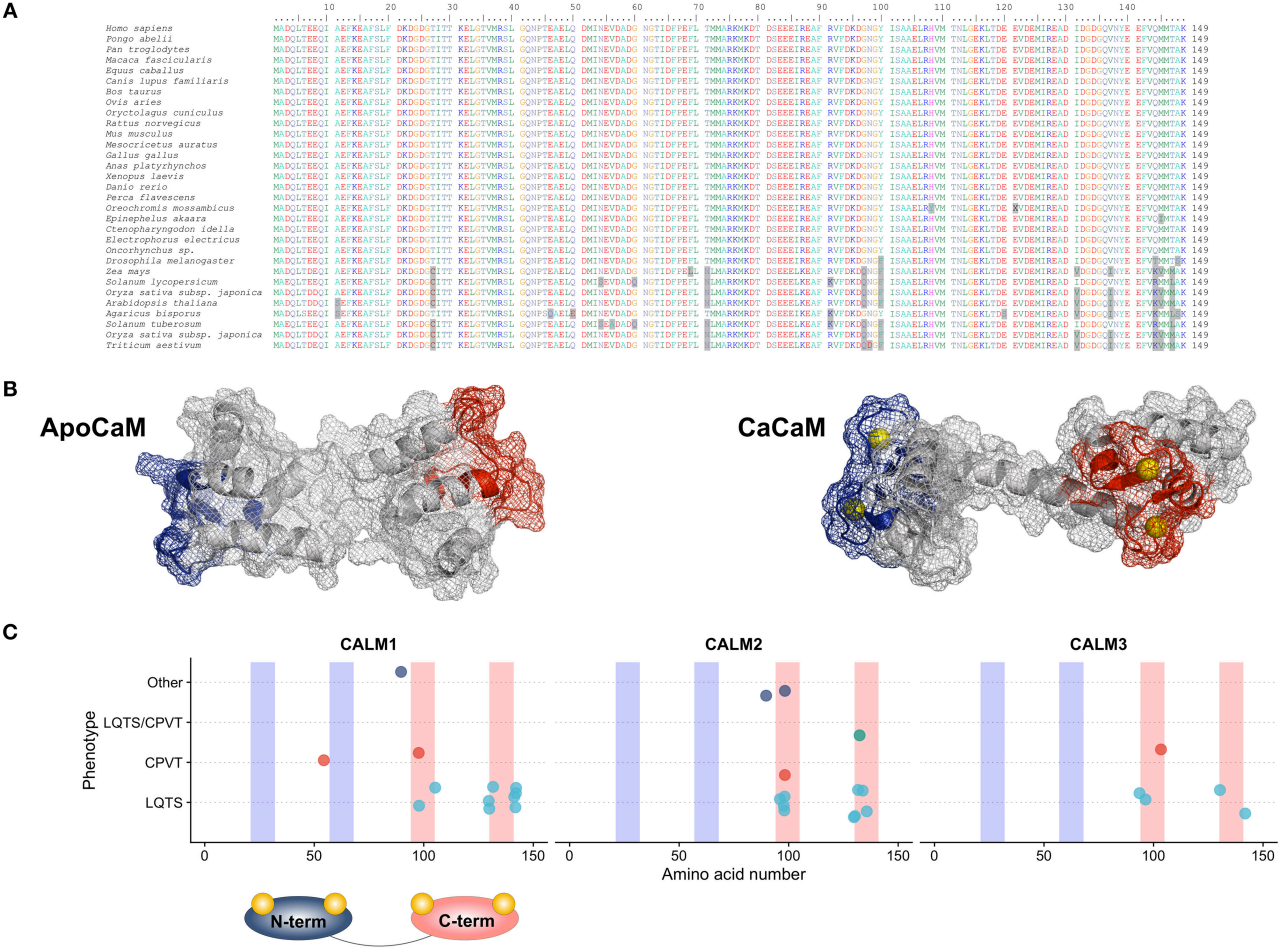
**(A)** Multiple species alignment of the CaM protein sequence showing full sequence conservation across vertebrates and an impressive sequence conservation across lower eukaryotes. **(B)** CaM protein structure in the ApoCaM **(left)** and Holo- or CaCaM conformation **(right)**. The N-terminal and C-terminal Ca^2+^ lobes are shown in blue and red, respectively. Ca^2+^ ions are shown in yellow. PDB structures entries: ApoCaM PDB ID: 1CFD; CaCaM PDB ID: 1CLL. **(C)** Genotype-phenotype correlation graphic representation of published CaM mutations, with respect to the gene and domain in which the mutations reside. C-terminal Ca^2+^-binding domains are shown in red and N-terminal Ca^2+^-binding domains are shown in blue.

Calmodulin in humans is indeed encoded by three different genes (*CALM1, CALM2* and *CALM3*; NG_013338.1, NG_042065.1 and NG_051331.1 RefSeqGenes, respectively), located on three different chromosomes (14q32.11, 2p21 and 19q13.32, respectively). Although several splice variants exist, only few transcripts contribute to the full-length CaM protein with the principal ones being NM_006888.4, NM_001743.5 and NM_005184.3, respectively. The three genes have a similar exon-intron structure, with 6 coding exons (14–16). The CaM protein generated from the translation of each of the three main transcripts has a length of 149 amino acids and an identical amino acid sequence. Its evolutionary importance is highlighted not only by the presence of three different genes located on three different chromosomes encoding the same protein, but also by the extent of conservation of its protein sequence that is full across vertebrates and very high across all eukaryotes (17) (Figure 1A).

Despite CaM's significant conservation, the respective *CALM* genes share only approximately 80% sequence identity among their coding regions, while they have no significant homology in their non-coding regions (14). This shows that the *CALM* genes have diverged early during evolution (14) and are not the result of gene duplications as is often the case with multigene families, while operating under a strict evolutionary control that aims to maintain their structural integrity.

Although CaM's genetic redundancy may partly serve its ubiquitous nature and support its pivotal role in several processes essential for cell survival and functioning, little is known about *CALM* genes' differential expression. Gene expression analyses have shown that in mammalian cells (16) and human hearts at the fetal, infant and adult stages of development (6), significantly higher transcriptional levels of *CALM3* than *CALM2* and *CALM1* are observed, with the latter being the least transcribed. Data from the Expression Atlas (18) and the Human Protein Atlas (19) indicate that all three CaM genes are ubiquitously expressed in the majority of tissues, with high and medium levels of expression found in the brain and heart, respectively. At the protein level, the relative contribution of each transcript is still unclear. Upon the initial identification of different cDNA clones, suggesting the existence of three different genes, it was hypothesized that one gene could be the housekeeping gene, while the other two could be differentially expressed under conditions of particular stimuli (14). This however seems not to be the case and all transcripts contribute toward CaM's overall expression (20), albeit unclear to which extent.

## Calmodulin's Structure

Human CaM is a small 149 amino acid protein with a length of 65 Angstroms (Å) that could be pictured in the shape of a dumbbell (21). It is comprised of two Ca^2+^-binding lobes at its N- and C-terminus which are linked by a flexible helix, each containing two EF-hand (helix-loop-helix) structural motifs with Ca^2+^-binding properties (Figure 1B). The lobes display cooperative Ca^2+^-binding properties (22), with faster Ca^2+^ binding at the N-terminal lobe and higher Ca^2+^-binding affinity at the C-terminal lobe (23), thus allowing CaM to function either as a rapid or slow Ca^2+^ sensor (24).

Within the cell, CaM may exist in multiple conformations that can be summarized in a six-state folding model, dependent on intracellular Ca^2+^ levels (25). Calmodulin folding and regulation have been evolutionally selected as robust key mediators of multiple Ca^2+^-based intracellular signals to control the activity of downstream targets in response to a broad range of intracellular Ca^2+^ concentration changes and different states of energetic stability (25). At low intracellular Ca^2+^ concentrations, the empty, Ca^2+^-free form (ApoCaM) prevails; increasing Ca^2+^ concentration leads to a progressive and cooperative occupation of the four Ca^2+^-binding sites until the saturated form (CaCaM or *HoloCaM*) is reached. Ca^2+^ binding and folding properties of each domain do influence both target binding and target regulation and confer to CaM the pliability to sense both local and global Ca^2+^ levels (24).

## Calmodulin's Interaction Network

Multiple cardiac ion channels and pumps are targeted and regulated by intracellular Ca^2+^ through Ca^2+^-sensing proteins, with CaM being the predominant and most widespread. This Ca^2+^-dependent regulation acts on multiple levels and involves, among others, sarcolemmal ion channels responsible for the L-type Ca^2+^ current (I_CaL_, Ca_v_1.2), the peak sodium current (I_Na_, Na_v_1.5), the slow delayed rectifier potassium current (I_Ks_, K_v_7.1), the inward rectifier potassium current (I_K1_, K_ir_2.1) and sarcoplasmic reticulum (SR) proteins such as the cardiac ryanodine receptors (RyR2) and phospholamban (PLB) (11). The regulation of these targets by CaM can be primary, through a direct association of CaM to its target, or mediated by the activity of Ca^2+^-CaM-dependent protein kinase II (CaMKII).

Different CaM pools do exist within the cell and the net result of this somehow unique protein distribution and equilibrium is that the vast majority (~99%) of CaMs appear to be already pre-bound to targets (26, 27), thereby assigning a buffering capacity to the remaining tiny fraction of the total CaM pool. This means that the three different CaM genes encode for identical CaM proteins that will be mostly bound to their targets. This scenario dramatically reduces the competition for target binding and constitutes a vulnerable substrate toward genetic mutations.

Under certain conditions, when the ApoCaM configuration is adopted, the N-terminal lobe has higher stability than the C-terminal lobe (25), which allows the binding/pre-association to important biological targets such as the Na_v_1.5 or Ca_v_1.2 channels in the ApoCaM form (28, 29). In particular, ApoCaM pre-association to Ca_v_1.2 is required since CaMs from the cytosolic bulk are unable to adequately access the binding site on Ca_v_1.2 during Ca^2+^ inflow (28); this property confers to the residual CaMs the ability to finely modulate substrate activities in response to local intracellular Ca^2+^ concentration changes (30, 31) with such a precision and velocity that would not be possible if Ca-CaM would have to be recruited from the cytosolic CaM bulk.

A certain degree of cytosolic CaM–level dependency is nevertheless maintained in the cells as a mechanism of Ca^2+^ homeostasis tuning other ion channels (32). Furthermore, according to modeling data published by Valeyev and coworkers, specificity and selectivity of CaM target regulation relies on target CaM-specific constants of dissociations and on the number of Ca^2+^ ions required for CaM-target complex activation (33), with some interactions requiring a preferential Ca^2+^ binding to the N-terminus (34), C-terminus or both.

Results by multiple groups have converged on deciphering the role of CaM's two main targets that underlie and define the main calmodulinopathy phenotypes, i.e., Ca_v_1.2 and RyR2, encoded by *CACNA1C* and *RYR2*, genes with a previously established role in LQTS and CPVT pathogenesis, respectively.

## Genetics of Calmodulinopathy

Twenty three mutations in one of the three CaM genes have been identified so far in thirty index calmodulinopathy patients with different arrhythmic phenotypes (6, 7, 9, 10, 35–43) (Table 1). The mutations thus far described have mainly arisen *de novo*, as demonstrated by parental genetic screening in 23 families. In only three cases the mutation was inherited (*CALM3*-p.A103V, *CALM1*-p.N54I, *CALM1*-p.F90L (9, 10, 35) and was shown to co-segregate with an arrhythmic phenotype in the respective families.

**Table 1:** List of published CaM mutations associated with arrhythmic phenotypes.

**Gene**	**Nucleotide change**	**Amino acid change**	**Protein domain**	**N. of probands**	**Associated phenotype**	**References**
*CALM1*	c.161A>T	p.N54I	inter-EF hand I-II linker	1	CPVT	(9)
*CALM1*	c.268T>C	p.F90L	inter-EF hand II-III linker	1	IVF	(10)
*CALM2*	c.268T>C	p.F90L	inter-EF hand II-III linker	1	SUD	(43)
*CALM3*	c.281A>C	p.D94A	EF-hand III	1	LQTS	(37)
*CALM3*	c.286G>C	p.D96H	EF-hand III	1	LQTS	(40)
*CALM2*	c.287A>T	p.D96V	EF-hand III	1	LQTS	(6)
*CALM1*	c.293A>G	p.N98S	EF-hand III	2	LQTS, CPVT	(9, 37)
*CALM2*	c.293A>G	p.N98S	EF-hand III	4	LQTS,CPVT,SUD	(36, 38, 43)
*CALM2*	c.293A>T	p.N98I	EF-hand III	1	LQTS	(38)
*CALM3*	c.308C>T	p.A103V	EF-hand III	1	CPVT	(35)
*CALM1*	c.314A>C	p.E105A	EF-hand III	1	LQTS	(42)
*CALM1*	c.389A>G	p.D130G	EF-hand IV	2	LQTS	(6)
*CALM2*	c.389A>G	p.D130G	EF-hand IV	1	LQTS	(41)
*CALM3*	c.389A>G	p.D130G	EF-hand IV	1	LQTS	(7)
*CALM2*	c.389A>T	p.D130V	EF-hand IV	1	LQTS	(41)
*CALM2*	c.394G>C	p.D132H	EF-hand IV	1	LQTS	(39)
*CALM1*	c.395A>T	p.D132V	EF-hand IV	1	LQTS	(39)
*CALM2*	c.396T>G	p.D132E	EF-hand IV	1	LQTS/CPVT Overlap	(38)
*CALM2*	c.400G>C	p.D134H	EF-hand IV	1	LQTS	(38)
*CALM2*	c.407A>C	p.Q136P	EF-hand IV	1	LQTS	(38)
*CALM1*	c.422A>G	p.E141G	EF-hand IV	1	LQTS	(41)
*CALM1*	c.426C>G	p.F142L	C-terminal region	3	LQTS	(6, 41)
*CALM3*	c.426T>G	p.F142L	C-terminal region	1	LQTS	(40)
Total	23	18		30		

All 23 mutations described are single nucleotide substitutions, leading to 18 distinct missense amino acid changes and are prevalently distributed in the *CALM1* (*n* = 8) and *CALM2* (*n* = 10) genes, while a few reside in the *CALM3* gene (*n* = 5). Interestingly, the majority of mutations (18/23, 78%) are localized in CaM's C-terminal Ca^2+^-binding domains (EF-hands III and IV) (Figure 1C), and especially in the specific residues directly involved in Ca^2+^ binding (16/23, 70%). Since the C-terminal EF-hands (III and IV) have been demonstrated to have a higher Ca^2+^-binding affinity than those in the N-terminus (I and II), calmodulinopathy's mutation distribution strongly highlights the importance of Ca^2+^-binding affinity for proper protein function and indicates specific topological domains that seem to be intolerant to genetic variation.

In support of the latter also come data on CaM genetic variation from the general population (genome aggregation database, gnomAD) (44). Not only thus far described calmodulinopathy-causative variants are absent from the general population, but also, the *CALM* non-synonymous genetic variants that are present (*n* = 29) have a different distribution within the CaM protein compared to the calmodulinopathy-causing mutations. In fact, the former are mainly localized outside the Ca^2+^-binding domains, in the EF-hand linkers and in the N- and C-terminal ends of the protein. In addition, the C-terminal EF-hands III and IV host recurrent calmodulinopathy-causing mutations, such as the p.N98S, p.D130G, and p.F142L, identified thus far in 6, 4 and 4 index cases, respectively, further highlighting the functional importance and intolerance to variation of these topological domains within the CaM protein.

## Calmodulinopathy Disease Mechanisms

**Figure 2:**
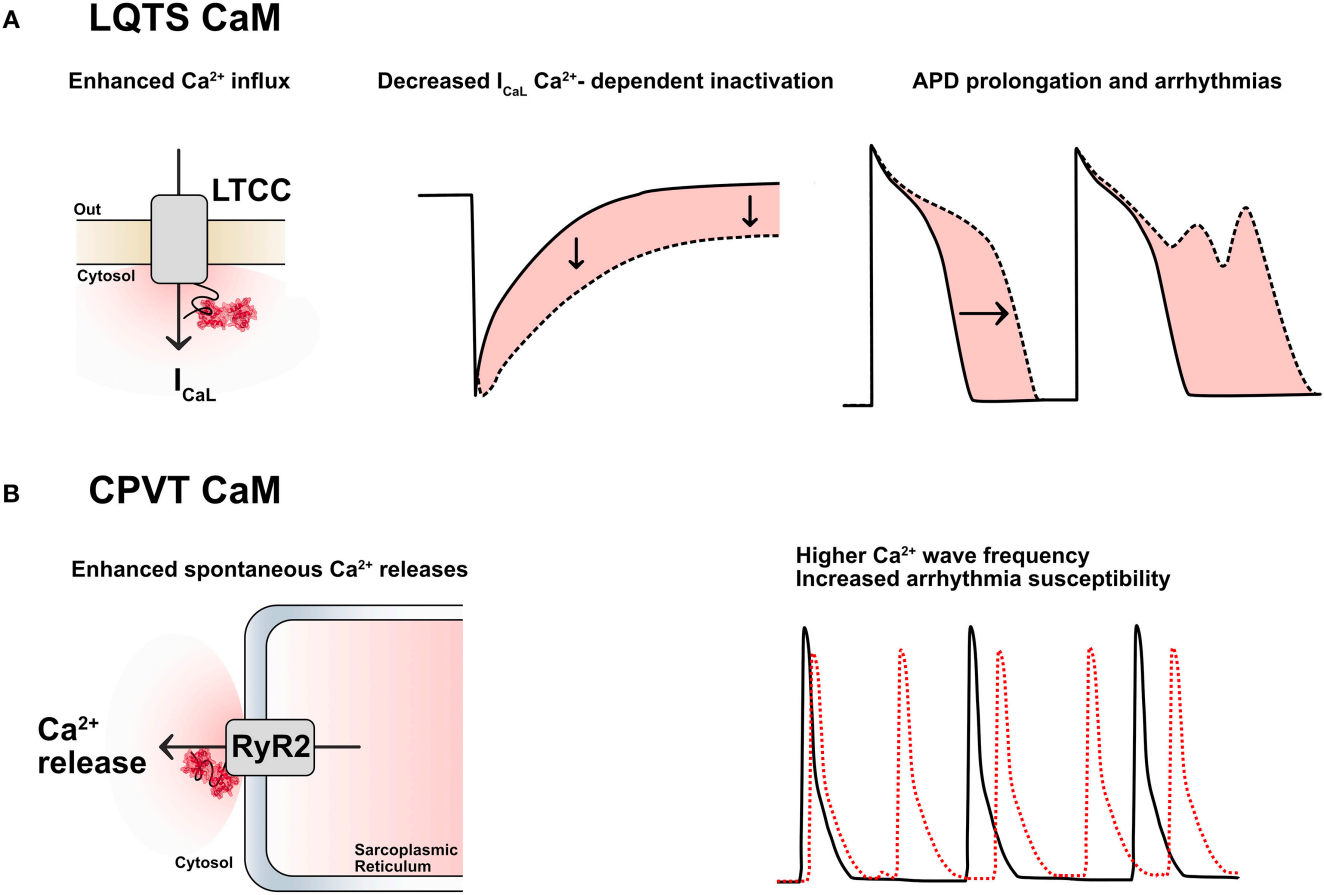
**(A)** LQTS-CaMs induce an increased Ca^2+^ influx via I_CaL_ by decreasing its Ca^2+^-dependent inactivation (CDI); this causes an enhanced Ca^2+^ influx, a consequent action potential duration (APD) prolongation and arrhythmias. LTCC: L-type calcium channels. **(B)** CPVT-CaMs promote Ca^2+^-release from the sarcoplasmic reticulum through the cardiac ryanodine receptors (RyR2). This leads to a higher frequency of Ca^2+^ waves and to a higher arrhythmia susceptibility, especially in the presence of catecholamines.

Among all CaM mutations thus far described (45), 11 have been functionally investigated *in vitro*, mostly by Ca^2+^-binding assays and/or electrophysiological studies. In our initial description of LQTS-associated calmodulinopathy (6) we provided preliminary evidence that the CaM mutations identified exhibited reduced affinity for Ca^2+^ and were thereby predicted to interfere with CaM's ability to transduce Ca^2+^-mediated signals. Diminished Ca^2+^-binding capacity had also been previously demonstrated by *in vitro* overexpression of mutant CaM in mammalian cardiomyocytes (CMs), which resulted in severe action potential prolongation, i.e., a cellular recapitulation of LQTS (46). We have further validated this reduced affinity for Ca^2+^ in the context of other LQTS-related CaM mutations (38, 39). Moreover, we have identified impaired Ca^2+^-dependent inactivation (CDI) of the L-type Ca^2+^ channel Ca_v_1.2- with concordant disruption of cellular Ca^2+^ homeostasis- as the prominent mechanism of LQTS-associated calmodulinopathy (39, 47) (Figure 2A). Our findings have been in accordance with those of other investigators.

Calcium-dependent inactivation is the mechanism by which conformational changes in CaM, driven by extracellular Ca^2+^ inflow through Ca_v_1.2, modulate part of the inactivation of the LTCCs. This mechanism acts as a negative feedback loop to precisely control the amount of Ca^2+^ entering the cardiomyocyte at each heartbeat and it is deranged in the presence of certain CaM mutations associated with an LQTS phenotype (LQTS-CaM), (Figure 2A). Multiple lines of evidence converge on the fact that LQTS-CaM mutations decrease CaM's affinity for Ca^2+^ and lead to an impaired CDI, resulting in an increased and uncontrolled Ca^2+^ inflow, action potential (AP) prolongation, QT interval prolongation and potentially lethal arrhythmogenic events. Conversely, other CaM mutations do not impair CaM's Ca^2+^-binding properties, but, rather, they strengthen CaM's binding affinity to RyR2, thus promoting the open conformation of RyR2 and interfering with its fine regulation. When dysfunctional interactions between CaM and RyR2 occur, e.g., due to mutations associated to a CPVT phenotype (CPVT-CaM), SR Ca^2+^ content, along with its feedback mechanism, are dysregulated, leading to premature and spontaneous Ca^2+^ releases from the SR and arrhythmogenic propensity (48) (Figure 2B).

**Figure 3:**
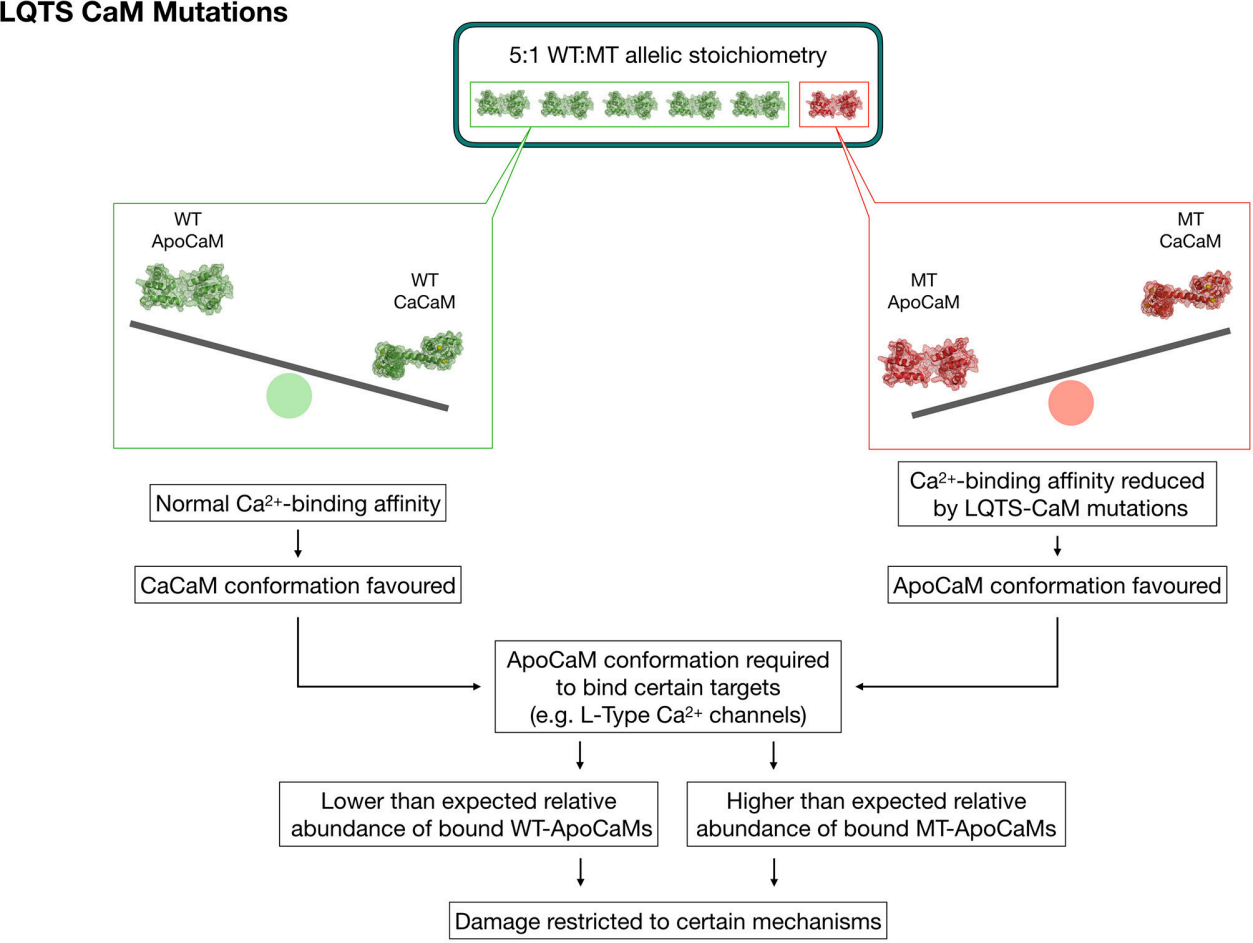
Graphic representation depicting a theory to explain the strong phenotypic effect and allelic dominance of CaM mutants in the setting of heterozygous LQTS-CaM mutations. The lower Ca^2+^ binding affinity of LQTS-CaMs creates a larger than expected pool of LQTS-ApoCaMs, i.e., the conformation that binds LTCCs, with a consequent higher-than-expected abundance of LQTS-CaM bound to LTCCs. Thus, LTCCs will have a high percentage of dysfunctional CaMs bound, which will significantly disrupt their activity despite the 5:1 WT:MT allelic stoichiometry.

The polyhedral nature of CaM's interaction network certainly hampers the identification of straightforward links between clinical manifestations of calmodulinopathy and its underlying molecular bases. Although several hypotheses have been formulated, many of which by our group (6, 49), and calmodulinopathy's main molecular mechanisms delineated, a generalized consensus and in-depth understanding of the reasons leading to such severe clinical phenotypes, are still pending. The current hypothesis to explain the malignancy of the disease manifestations mainly relies on two mechanisms which might synergistically concur to shape the disease phenotype (Figure 3). Considering that 99% of CaMs are already pre-bound to their targets, this logically implies that also mutant CaMs will be bound to targets. However, this hypothesis alone, especially in the presence of a plethora of downstream mediators, may not fully explain the funneling of clinical manifestations into two main phenotypes, sometimes even in the presence of identical mutations.

The reason may partly rely also on a second mechanism, which involves the collateral consequences of ApoCaM's molecular interaction with its targets. Given that CaM pre-association to LTCC does not require Ca^2+^, it is reasonable to speculate that mutations impacting on the Ca^2+^-binding affinity of CaM should not theoretically alter ApoCaM's substrate-binding ability *per se*, but, on the contrary, they may indirectly enable it since mutant CaMs have a lower affinity for Ca^2+^ in their C-terminus lobe and thus are more likely to be found in the ApoCaM form. The data generated by the Yue lab (50) demonstrated, through elegant FRET assays, that LQTS-CaMs have at least an equal affinity for LTCC in the ApoCaM form, with some LQTS-CaM mutations exhibiting even a higher affinity than WT-CaMs. Therefore, targets that require a pre-association of ApoCaM will be preferentially bound to LQTS-CaM mutants, which would explain both the fact that some targets seem unaffected by these mutations, as well as the dominant expression of the pathogenic phenotype despite the wild type-favoring (5:1) allelic balance (Figure 3).

## Induced Pluripotent Stem Cell-Derived Cardiomyocyte Platforms for Calmodulinopathy Modeling *in vitro* and Testing of Therapeutic Approaches

Most of the studies that have aimed to characterize the relative effects of CaM mutations have used heterologous expression systems such as transient expression of CaM mutants in HEK293 (48, 50), tsA201 cells (41, 47), mammalian CMs (35, 41, 48, 50), or human induced pluripotent stem cells (hiPSCs) from healthy donors (39). Although these studies have provided insight into the prominent pathophysiological mechanisms of calmodulinopathy, they have had the major inherent limitation of not being able to faithfully recapitulate the *in vivo* effects by simply not respecting the native stoichiometry of mutant and wild-type CaM proteins. Having three different genes encoding the same CaM protein implies that in the presence of a mutation in a heterozygous state, there is a 5:1 wild-type:mutant allelic stoichiometry, with the quantitatively underrepresented mutant CaM still being able to confer its devastating effects. The redundancy of the CaM genes hampers also the generation of transgenic animal models, with diverse CaM gene copy numbers among species representing a substantial caveat for *in vivo* disease modeling.

In order to provide a more profound understanding of the effects conferred by CaM mutations in their native CM environment, multiple groups, including ours, generated hiPSCs from patients with CaM mutations (20, 49, 51). With the confidence of working in a physiologically relevant experimental model, CaM mutation rescue strategies have been recently attempted with multiple approaches. Limpitikul and coworkers used an elegant CRISPRi gene silencing approach to precisely suppress the transcription of the mutant *CALM2*^D130G^ allele, allowing a complete rescue of the LQTS phenotype (20). Yamamoto and coworkers, instead, followed a gene editing approach to selectively knockout the *CALM2*^N98S^ allele (51). Although not currently feasible in humans, this procedure demonstrated that the *CALM* loci can be realistic targets for gene correction approaches. Finally, Rocchetti and coworkers rescued the pathogenic LQTS phenotype caused by a *CALM1*^F142L^ mutation investigated in detail in hiPSC-CMs from a calmodulinopathy patient (6) by blocking I_CaL_ with verapamil (49). The field potential (FP) duration, measured with MEA, was rescued with acute exposure to verapamil, holding promise for a rapid implementation of this pharmacological approach in the clinical setting. This observation was immediately translated by Webster and coworkers in clinical practice and in a single calmodulinopathy patient (52) verapamil showed some promising results.

Overall, these studies demonstrated the importance and feasibility of modeling calmodulinopathy in the setting of its native environment, provided insight into the pathophysiological mechanism underlying the respective life-threatening arrhythmias and validated calmodulinopathy patient-derived iPSC-CMs as a platform for precision medicine investigations.

## Calmodulinopathy Phenotypes and Clinical Features

The rapid emergence of the genetic discoveries related to calmodulinopathy has eclipsed the ability of the field to ascertain and compare in depth all the individual clinical features among different subjects thus far described in the literature. This has naturally led to the “lumping” of cases under existing clinical “umbrella” terms. Indeed, CaM mutations have been associated with a spectrum of arrhythmic phenotypes, including LQTS, CPVT, IVF, as well as LQTS/CPVT overlap and Sudden Unexplained Death (SUD) (6, 7, 9, 10, 35–43). From the thus far available descriptions, however, it seems to emerge that there is a novel constellation of symptoms, arrhythmia types, ECG features and response to therapy that are characteristic of calmodulinopathy as a clinical entity, closely-related, but nevertheless distinct, from other arrhythmia syndromes.

To this end, we have recently established an international registry of subjects with CaM mutations (ICalmR) (45), currently enrolling patients from overall 15 countries, in order to conduct a thorough ascertainment of the clinical spectrum, genotype-phenotype relationship, and treatment responses of these patients. In order to further aid the enrollment of new cases and facilitate the discussion among experts, ICalmR has become a member of the European Reference Network on rare diseases of the heart (53), while, recently, the registry has been made available to the European Commission for online data submission and patient enrollment, according to EU requirements.

From the descriptions thus far available in the literature, the most prevalent phenotype is LQTS, observed in 73% (22/30) of index cases. All LQTS-associated CaM mutations are localized within CaM's EF-hands III (*n* = 7) and IV (*n* = 9) or in the C-terminal region (*n* = 2). The second most frequent arrhythmic phenotype observed is CPVT, with 4 index cases carrying the *CALM1*-p.N54I, *CALM1*-p.N98S, *CALM2*-p.N98S, or *CALM3*-p.A103V mutations. Much less represented phenotypes are LQTS/CPVT overlap (1 case, *CALM2*-p.D132E), IVF (1 case, *CALM1*-p.F90L), and SUD (2 cases, *CALM2*-p.F90L, *CALM2*-p.N98S). Interestingly, particular hotspot mutations, such as the p.N98S mutation, may give rise to diverse arrhythmic phenotypes (LQTS, CPVT, SUD), regardless of the gene in which the nucleotide substitution resides, whilst others, such as the p.D130G and p.F142L, always cause definite LQTS.

Irrespective of the associated phenotypes, a common feature of all CaM mutations identified so far is the extreme severity of disease manifestations and early occurrence with almost half of subjects having a perinatal presentation (28th week of gestation to 28th postnatal day) (12/28, 43%, with the exclusion of 2 SUDs), mainly among those with an LQTS phenotype. The latter often show 2:1 AV block, T wave alternans and QTc values above 550 ms (45).

As in other inherited arrhythmogenic conditions, SCD can be the first manifestation of the disease, but unfortunately SCD can also occur after diagnosis and establishment of antiarrhythmic therapies. Indeed, to provide some examples, in two patients, both carrying the *CALM1*-p.F142L mutation, the diagnosis of LQTS was made just after birth and beta-blocker therapy was immediately started. Later on, a pacemaker was implanted. Nevertheless, they both later died suddenly, at 2 and 1 years of age (41). Another LQTS subject, carrying the *CALM2*-p.Q136P mutation, came to medical attention at 8 years of age after an episode of syncope associated with a prolonged period of unconsciousness. At that time, she had a prolonged QTc of 500 ms with ventricular bigeminy, she was put on nadolol, but she died suddenly during a dancing competition at 11 years of age (38).

Unfortunately, half of calmodulinopathy patients will experience SCD (45) and may survive only if, often by chance, appropriate resuscitation will be provided in due time. Among those who will survive, many may end up neurologically compromised. Of note, in our original description of LQTS-associated calmodulinopathy (6), all patients presented with some neurodevelopmental/neurological features. However, it is still unclear whether neurological impairment may be an inherent feature of the disease spectrum, since CaM is also highly expressed in the brain, or such an impairment may be only the secondary result of multiple resuscitated cardiac arrests causing brain injury. By means of studying the disease systemically and by jointly examining a larger number of cases through the ICalmR (45), some light may be shed on whether CaM's non-cardiac expression may also give rise to concomitant non-cardiac phenotypes in the setting of calmodulinopathy.

## Calmodulinopathy Therapy

Calmodulinopathy is a severe condition for which effective therapies are currently lacking. From the clinical data and descriptions available thus far in the literature, and from our own clinical experience (45), commonly used anti-arrhythmic therapies and procedures (i.e., left cardiac sympathetic denervation) largely fail to treat these young patients. Indeed, β blocker therapy- the mainstay treatment for LQTS and CPVT- seems to offer minimal benefit at controlling the life-threatening arrhythmias, while other antiarrhythmics, such as sodium channel blockers, have also not produced promising results (6, 52). Ca^2+^ channel blockade may seem a rational therapeutic option since impaired CDI of the Ca^2+^ channel Ca_v_1.2 is the prominent underlying mechanism of LQTS related to CaM mutations (20, 49, 51). However, the Ca^2+^ antagonist verapamil, despite some positive results in one case (52), largely fails to prevent cardiac event recurrences in other clinical cases (6, 45). This may be attributed to the fact that verapamil principally targets peak calcium current rather than modulating channel inactivation (52).

Although implantable cardioverter defibrillators (ICD) would be the treatment of choice for any patient surviving SCD due to VF (54), this type of intervention in these young patients imposes almost impossible clinical dilemmas. On one hand, the risk of complications is higher than in adults, but, on the other hand, these children are at very high risk of dying suddenly or having neurological sequelae due to brain injury secondary to multiple resuscitated cardiac arrests. Unfortunately, we have examples showing a premature death in both scenarios (i.e., death related to ICD complications or death related to VF when the ICD was not implanted).

These real life examples highlight the urgent need to identify appropriate management schemes and therapies for life-threatening calmodulinopathy.

## Conclusion

Genetic mutations in any of the 3 genes encoding the ubiquitous Ca^2+^ signaling protein CaM cause calmodulinopathy, a recently identified severe arrhythmogenic entity, affecting very young individuals. Although more arrhythmic phenotypes have been associated to CaM mutations, LQTS is the predominant one, with CDI of the L-type Ca^2+^ channel Ca_v_1.2 and disruption of cellular Ca^2+^ homeostasis being the main underlying mechanism. To date, no therapies exist that may effectively treat the life-threatening arrhythmias and prevent SCD occurrence, while ICD implantation in these young patients is frequently associated with complications.

State-of-the-art technologies such as patient-derived iPSC-CMs have been successfully thus far employed for calmodulinopathy modeling, not only providing insight into the mechanistic bases but also showing promising results for future precision medicine investigations, e.g., through gene correction approaches.

Systematic clinical evaluation of a large number of patients and identification of new cases prospectively in combination with the most recent technological advancements in hiPSC-CM technology, pharmacological screenings (55), and tridimensional approaches (56, 57), hold promise in identifying effective therapeutic strategies for this devastating disease.

## Author Contributions

M-CK wrote the manuscript and drew the protein alignments. LS wrote the manuscript and drew the figures. AG wrote the manuscript. BB, CR, GP, and AZ critically reviewed the manuscript. LC wrote and critically reviewed the manuscript.

### Conflict of Interest Statement

The authors declare that the research was conducted in the absence of any commercial or financial relationships that could be construed as a potential conflict of interest.
